# Supplementary material for: Complex Physical Structure of Complete Mitochondrial Genome of Quercus acutissima (Fagaceae): A Significant Energy Plant
Source: Genes (Basel). 2022 Jul 24;13(8):1321. doi: 10.3390/genes13081321 (PMC9331829; doi:10.3390/genes13081321)
Supplement: Supplementary file 1 [file genes-13-01321-s001.zip › genes-1826170-supplementary.pdf]

Table S1. Gene composition in the *Q. acutissima* mitogenome.

Group of genes	Name of genes
ATP synthase	*atp*1，*atp*4，*atp*6，*atp*8，*atp*9
NADH dehydrogenase	*nad*1，*nad*2，*nad*3，*nad*4，*nad*4L，*nad*5，*nad*6，*nad*7，*nad*9
Cytochrome c biogenesis	*cob*
Ubiquinol cytochrome c reductase	*ccm*B，*ccm*C，*ccm*FC，*ccm*FN
Cytochrome c oxidase	*cox*1，*cox*2，*cox*3
Maturases	*mat*R
Transport membrane protein	*mtt*B
Large subunit of ribosome	*rpl*10，*rpl*2，*rpl*5
Small subunit of ribosome	*rps*10，*rps*12，*rps*3，*rps*4
Succinate dehydrogenase	*sdh*3
Ribosome RNA	*rrn*18，*rrn*26，*rrn*5
Transfer RNA	*trn*C-GCA，*trn*D-GUC，*trn*E-UUC (×2)，*trn*F-GAA，*trn*G-GCC，*trn*H-GUG，*trn*K-UUU，*trn*M-CAU (×3)，*trn*N-GUU，*trn*P-UGG (×2)，*trn*Q-UUG，*trn*S-GCU，*trn*S-UGA，*trn*V-GAC，*trn*W-CCA，*trn*Y-GUA

Table S2. RSCU for each amino acid pair in the *Q. acutissima* mitogenome.

Amino	Codon 1 RSCU	Codon 2 RSCU	Codon 3 RSCU	Codon 4 RSCU	Codon 5 RSCU	Codon 6 RSCU
Ala	GCU<br>1.601	GCA<br>0.984	GCC<br>0.903	GCG<br>0.511		
Arg	AGA<br>1.443	CGA<br>1.28	CGU<br>1.171	CGG<br>0.781	AGG<br>0.717	CGC<br>0.608
Asn	AAU<br>1.337	AAC<br>0.663				
Asp	GAU<br>1.358	GAC<br>0.642				
Cys	UGU<br>1.169	UGC<br>0.831				
End	UAA<br>1.406	UGA<br>1.031	UAG<br>0.563			
Gln	CAA<br>1.488	CAG<br>0.512				
Glu	GAA<br>1.402	GAG<br>0.598				
Gly	GGA<br>1.42	GGU<br>1.27	GGG<br>0.759	GGC<br>0.552		
His	CAU<br>1.508	CAC<br>0.492				
Ile	AUU	AUC	AUA			

	1.309	0.857	0.834			
Leu	UUA	CUU	UUG	CUA	CUC	CUG
	1.462	1.234	1.168	0.929	0.639	0.567
Lys	AAA	AAG				
	1.184	0.816				
Met	AUG					
	1.000					
Phe	UUU	UUC				
	1.139	0.861				
Pro	CCU	CCA	CCC	CCG		
	1.43	1.154	0.8	0.616		
Ser	UCU	UCA	AGU	UCC	UCG	AGC
	1.355	1.123	1.039	1.019	0.819	0.645
Thr	ACU	ACA	ACC	ACG		
	1.366	1.025	1.025	0.584		
Trp	UGG					
	1.000					
Tyr	UAU	UAC				
	1.521	0.479				
Val	GUU	GUA	GUG	GUC		
	1.211	1.179	0.837	0.773		
